# Effectiveness of bilingual health education on obesity and dietary behaviors in Mongolian adolescents: a six-month intervention study

**DOI:** 10.3389/fpubh.2026.1787774

**Published:** 2026-06-23

**Authors:** Hanhua Sai, Hailing Li, Nan Zhang, Huijuan Liang, Zhaligahu Xiao, Fang Tang, Shaoqiong Lu, Rihan Ao, Wudelehu Wu

**Affiliations:** 1 Inner Mongolia Medical University, Hohhot, China; 2 Student Affairs Department, Hohhot Minzu College, Hohhot, China; 3 The Department of Endocrinology, Inner Mongolia Autonomous Region People's Hospital, Hohhot, China

**Keywords:** agro-pastoral areas, bilingual health education, BMI *Z*-score, Mongolian adolescents, obesity

## Abstract

**Background:**

Obesity among adolescents has become a global public health concern. Previous studies have shown that multidimensional interventions, such as school-based programs, health education, and guidance on diet and physical activity, effectively improve obesity in adolescents. However, there is a lack of research on bilingual health education interventions in minority language environments in rural and pastoral areas of China. Based on a 2023 field survey of students from three Mongolian middle schools in Ar Horqin Banner, Chifeng City, Inner Mongolia, this study evaluated the effect of a 6-month Mongolian–Chinese bilingual health education intervention on obese adolescents' BMI *Z*-scores, dietary behaviors, and biochemical indicators (fasting blood glucose, insulin, and blood lipids).

**Methods:**

Baseline data, including height, weight, and a dietary behavior questionnaire, were collected from 2,210 Mongolian middle school students, of whom 155 obese students were screened out. A 6-month bilingual health-education intervention was implemented for the 155 obese students, with content covering guidance on a rational diet, cultivation of healthy behaviors, and moderate exercise training. Before and after the intervention, the participants underwent height and weight measurements, fasting blood sampling, and completed questionnaires. This study evaluated the intervention effects by examining changes in BMI *Z*-scores, dietary behaviors, and glucose and lipid-related biomarkers (including fasting blood glucose, insulin, total cholesterol (TC), low-density lipoprotein cholesterol (LDL-C), high-density lipoprotein cholesterol (HDL-C), and triglycerides (TG)) among obese students pre- and post-intervention.

**Results:**

Among 2,210 adolescents, the prevalence of overweight and obesity was 13.12 and 7.01%, respectively (combined 20.13%). These rates were significantly higher among males and junior high school students (*P* < 0.001). The obesity rate is higher in males than in females (OR = 1.883, 95%CI: 1.338 ~ 2.650). Adolescent obesity was significantly associated with parental obesity (OR = 1.847, 95% CI: 1.172 ~ 2.996), specific dietary behaviors (e.g., frequent midnight snacking), reduced physical activity (OR = 2.262, 95% CI: 1.254 ~ 4.081), prolonged sleep duration (OR = 1.622, 95% CI: 1.148 ~ 2.291), and severe body dissatisfaction (*P* < 0.05). A 6-month health education intervention conducted on 111 adolescents with obesity (44 cases were missing due to various reasons) improved their health awareness, dietary choices, exercise habits, and sleep patterns (*P* < 0.05). Additionally, compared with baseline and 6 months, changes in BMI *Z*-scores in males were statistically significant at 3 months (*P* < 0.05). The intervention significantly improved metabolic profiles: fasting blood glucose, total cholesterol, and LDL-C levels were significantly reduced (all *P* < 0.05), and the prevalence of abnormal LDL-C and total cholesterol dropped to 0% at the 6-month follow-up.

**Conclusion:**

Bilingual health education effectively improved dietary behavior and metabolic indicators in adolescents with obesity. Although the 6-month intervention did not significantly alter overall BMI *Z*-scores, the short-term decrease observed exclusively in males suggests a potentially beneficial effect on weight management. Ultimately, sustainable obesity prevention and control require collaborative efforts from the government, schools, and families.

## 1 Introduction

In recent years, the prevalence of overweight and obesity among adolescents has been high, and adolescent obesity has become a global public health challenge. According to reports, from 2000 to 2023, the overall prevalence of obesity among adolescents in 154 countries or regions worldwide was 8.5%, with one in every five adolescents being overweight (1). In China, the number of overweight and obese individuals has rapidly increased over the past 40 years. Between 2015 and 2019, the overweight rate among adolescents under the age of six was 6.8%, and the obesity rate was 3.6%. Among adolescents aged 6–17 years, the overweight and obesity rates were 11.1 and 7.9 %, respectively (2).

There are many harms associated with adolescent overweight or obesity. A direct consequence of the high incidence of childhood obesity is the increased risk of nonalcoholic fatty liver disease (NAFLD), which has become the main cause of chronic liver disease among adolescents (3, 4). Moreover, adolescent females who are overweight and/or obese have a higher probability of developing polycystic ovary syndrome (PCOS) than adults (5). In addition, obesity can lead to metabolic diseases such as dyslipidemia, hypertension, and diabetes, which seriously affect quality of life and longevity (6, 7). Studies have reported that BMI is linearly associated with the risk of 18 site-specific cancers, including malignant melanoma, leukemia, and multiple myeloma (8). Simultaneously, research indicates that severe childhood obesity is closely related to obesity in adulthood and the risk of multiple chronic diseases, such as diabetes, cancer, and cardiovascular disease, all of which are closely associated with overweight or obesity during adolescence (9, 10).

Research has indicated that from the age of 5 years, 40% of adolescents with obesity can already exhibit elevated parameters of insulin resistance, and insulin resistance in adolescents with obesity doubles the risk of future deterioration of blood sugar levels. Indicators based on fasting insulin are better predictors of future blood sugar deterioration than blood sugar levels alone, which helps stratify the risk profiles of adolescents with obesity and thus guide targeted prevention and intervention strategies for patients (11). A medical survey conducted among 1,112 adolescents aged 13–18 years in China found that all lipid profiles were closely related to metabolic syndrome, with the lipid accumulation product (LAP) index having the strongest association with metabolic syndrome (12). Studies have found that the average blood sugar levels of overweight and adolescents with obesity are higher than those of non-adolescents with obesity (13). Early identification of overweight and obesity can help improve the lifelong health of patients (14).

Inner Mongolia is the core region inhabited by ethnic Mongolians in China, where adolescents commonly use both Mongolian and Chinese across family, community, and school settings. Although Mongolian students typically possess basic Chinese proficiency, they process professional health knowledge, nutritional terminology, and behavioral guidance more accurately and demonstrate higher adherence when it is delivered in their native language. Previous health interventions for this population have predominantly relied on a Chinese-only approach, which often compromises effectiveness due to language barriers and inadequate cultural adaptation. Currently, there is a lack of empirical evidence regarding health promotion strategies that are tailored to bilingual ethnic minority groups. To address this gap, this study employs a bilingual Mongolian–Chinese health education model. This approach maintains scientific rigor while respecting linguistic habits and cultural backgrounds, aiming to improve the accessibility and acceptance of obesity-related health information, standardize dietary behaviors, and ultimately optimize intervention effectiveness among Mongolian adolescents.

This study investigated rural Mongolian adolescents using a short-term prospective intervention. The main objective of this study was to evaluate the impact of a 6-month bilingual health education intervention in Mongolian and Chinese on the BMI *Z*-score of adolescents. The secondary objective was to assess metabolic-related indicators (fasting blood glucose, fasting insulin, and blood lipids) and behavioral changes in obese adolescents. The study proposes the following research hypothesis: through bilingual health education in Mongolian and Chinese, dietary and exercise behaviors can be improved, the BMI *Z*-score of adolescents with obesity can be reduced, and the metabolic-related indicators can be significantly improved. This study established a multidisciplinary bilingual team of Mongolian and Chinese to provide culturally consistent health education to Mongolian adolescents in rural areas. By quantifying the intervention effects through behavioral and biomedical indicators, this study provides empirical evidence for obesity prevention and control strategies in ethnic minority regions with diverse languages.

## 2 Materials and methods

### 2.1 Study population

This cross-sectional survey used cluster sampling. All students in the 7th, 8th, 10th, and 11th grades from three Mongolian middle schools in Arukorqin Banner, Chifeng City, Inner Mongolia (Tianshan Mongolian Middle School of Arukorqin Banner, Tianshan Fifth Middle School of Arukorqin Banner, and Tianshan Second Middle School of Arukorqin Banner) were selected for the survey between March and April 2023. This study included 2,210 middle school students from ethnic minorities aged 11 to 18 years. Individuals with type 1 diabetes, secondary hypertension, thyroid dysfunction, severe liver or kidney disease, hypothalamic-pituitary-adrenal axis disorders, or long-term glucocorticoid use were excluded. This study was approved by the Ethics Committee of Inner Mongolia Medical University (Approval No. YKD202402149). Given that all participants were adolescents aged 11 to 18 years, written informed consent was obtained from their parents or legal guardians prior to their inclusion in the study. Additionally, written assent was obtained from all participants themselves after they were provided with age-appropriate explanations of the study purpose, procedures, potential risks, and their right to withdraw at any time without consequence. During data analysis, all data were de-identified and labeled only with ID numbers. They were kept strictly confidential and used solely for the purposes of this research.

**Figure 1:**
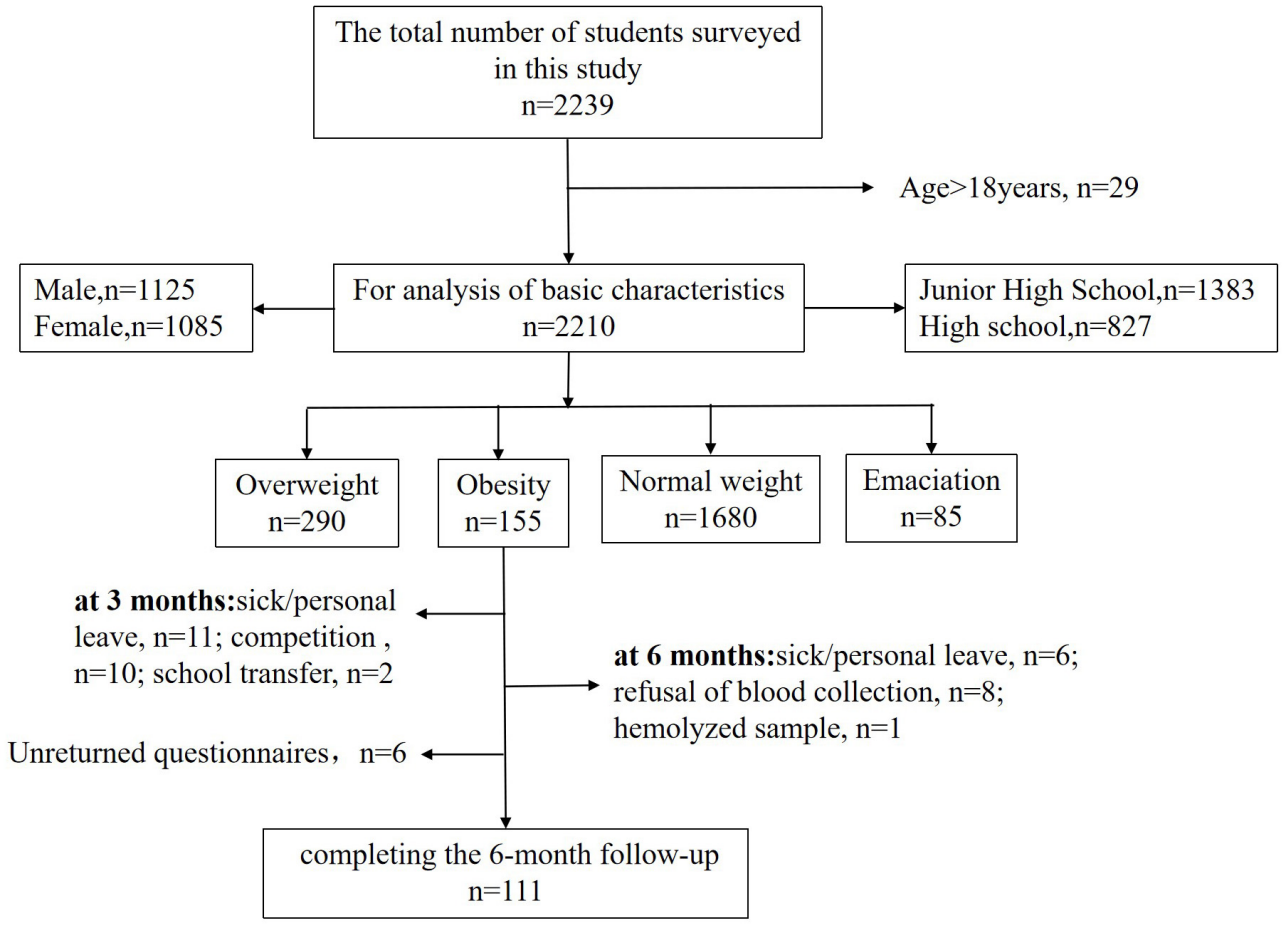
Flow diagram of participants.

According to the World Health Organization (WHO) growth standard criteria for overweight and obesity, 155 obese students who met these standards were identified as the target group for the health education intervention. However, during the follow-up intervention period, several participants were lost to follow-up due to sick leave, competition participation, refusal to provide biological samples, and other reasons. As a result, complete data from 111 participants were ultimately included in the analysis of BMI *z*-scores and blood test parameters. The flow diagram of study participants is shown in Figure 1.

### 2.2 Research methods

#### 2.2.1 Survey of overweight/obesity prevalence

A standard weight scale and height-measuring instrument were used to measure the students' height and weight, and the body mass index (BMI) was calculated as weight (kg)/height^2^(m^2^). All BMI *Z*-scores were calculated using the R packages “anthro” and “anthroplus” following the World Health Organization (WHO) growth standard, and converted to age-adjusted standardized *z*-scores (BMI *z*-score) using the sex-specific World Health Organization (WHO) BMI reference for adolescents aged 5–19 (15). BMI *Z*-score groups were defined as underweight (BMI *Z*-score < -2), normal weight (−2 < BMI *Z*-score < 1), overweight (1 < BMI *Z*-score ≤ 2), and obesity (BMI *Z*-score > 2).

Given the constraints of personnel and implementation resources, this study balanced intervention fidelity with the urgent needs of adolescents with obesity. Consequently, we prioritized adolescents with confirmed obesity and higher baseline health risks (*n* = 155) as the core intervention targets, aiming to maximize the effectiveness of the intervention under limited resources.

#### 2.2.2 Questionnaire survey of lifestyle and dietary behaviors

A self-designed lifestyle and dietary behavior questionnaire was used to assess the daily amount of exercise, dietary habits (e.g., intake frequency of staple foods, meat, vegetables, and fruit), and snack intake.

##### Questionnaire development and validity testing

The questionnaire items were generated from a literature review and three rounds of expert consultations. A pilot test was conducted with 30 students with similar characteristics. The Cronbach's α coefficient of the questionnaire was 0.83, and the content validity index at the scale level (S-CVI) was 0.87. These results support the validity of the questionnaire. Trained investigators guided the students in completing the questionnaire to ensure valid questionnaire responses.

#### 2.2.3 Health education intervention

The bilingual health education intervention was delivered by a multidisciplinary team using the following structured approach.

##### 2.2.3.1 Intervention team composition

A specialized multidisciplinary team was formed, comprising three endocrinologists (responsible for delivering medical theory), two nutritionists (responsible for dietary guidance), and three endocrinology nurses (responsible for on-site coordination and follow-up management). Crucially, all the team members were proficient in both Mongolian and Chinese.

##### 2.2.3.2 Intervention frequency and duration

The intervention consisted of two centralized group sessions conducted once every 3 months over a total observation period of 6 months.

##### 2.2.3.3 Core intervention content

*Obesity awareness*. Educating students on the health risks of obesity (e.g., metabolic disorders, psychological impacts, and musculoskeletal issues) and its contributing factors (genetics, environment, and dietary habits).

*Dietary management*. Introducing the Chinese Food Pagoda, teaching the calculation of daily caloric needs, and outlining macronutrient distribution. Special emphasis was placed on adapting traditional Mongolian diets (e.g., milk tea, beef, mutton, and dairy products) to healthier alternatives.

*Physical activity prescription*. Recommending age-appropriate aerobic exercises (e.g., brisk walking, swimming, jump rope), with specific targets of ≥5 days per week, ≥30 min per session, at moderate intensity.

##### 2.2.3.4 Bilingual educational modalities (Mongolian–Chinese)

*Multimedia lectures*. Lectures delivered orally in Mongolian, accompanied by PowerPoint presentations featuring Chinese subtitles and illustrated infographics.

*Supplementary materials*. Distribution of bilingual (Mongolian–Chinese) illustrated health education brochures.

*Video-based education*. Continuous looping of short, scientifically grounded nutritional videos featuring Mongolian dubbing and Chinese subtitles in the classroom.

*Interactive group discussions*. Students were divided into small groups (6–8 members) to discuss topics in Mongolian, such as strategies to reduce high-fat/high-salt snack consumption and how to balance school lunches. Each group was facilitated by a member of the medical team.

#### 2.2.4 Biochemical index testing

##### 2.2.4.1 Observation indicators

Health records were established, and key indicators were documented, such as height, weight, BMI *Z*-scores, waist circumference, blood pressure (systolic and diastolic), and measurements of fasting blood glucose (FBG), fasting insulin (FINS), and blood lipid levels. Blood lipids include triglycerides (TG), total cholesterol (TC), low-density lipoprotein cholesterol (LDL-C), and high-density lipoprotein cholesterol (HDL-C) levels. Height and weight measurements were conducted in accordance with the standardized requirements outlined in the “National Student Physical Fitness Health Survey and Verification Manual of National Student Physical Fitness Standards,” performed by professionally trained personnel.

##### 2.2.4.2 Detection methods

(1) FBG was measured using a Beckman Coulter AU680 automatic biochemical analyzer (Beckman Coulter, USA) with the hexokinase method; (2) FINS was measured using an Abbott I2000SR luminescence analyzer (Abbott, USA) by chemiluminescence; and (3) TG, TC, LDL-C, and HDL-C levels were measured using a Beckman Coulter AU680 automatic biochemical analyzer (Beckman Coulter, USA) with an immunoturbidimetric method. Dyslipidemia was defined according to the Chinese Guidelines for Lipid Management (2023) (16).

##### 2.2.4.3 Relevant index calculation formulas

(1) Homeostasis Model Assessment of Insulin Resistance (HOMA-IR) = FBG (mmol/L) × fasting insulin (μU/mL) / 22.5.
(2) Fasting Triglycerides and Glucose Index (TyG) = Ln (TG mg/dL × FBG mg/dL / 2) (17).

#### 2.2.5 Quality control

We developed a detailed data collection manual, standardized measurement instruments/protocols, and provided uniform training to the surveyors. During data collection, on-site verification and logical checks were performed to ensure accuracy. Data were independently double-entered, discrepancies were detected, and reconciled using EpiData.

### 2.3 Statistical analysis

Continuous variable distribution normality was verified via Kolmogorov–Smirnov testing. Parametric data are reported as mean ± standard deviation with between-group comparisons using the Student's *t*-test. Nonparametric variables are presented as medians (interquartile ranges) and were analyzed using Mann–Whitney *U*-tests. One-way analysis of variance (ANOVA) was used to compare differences in BMI *Z*-scores and biochemical indicators at different time points. The least significant difference (LSD) test was used as a *post hoc* test for results with statistically significant differences after One-way ANOVA. For non-normally distributed data, the Friedman test was used, with pairwise comparisons performed using the Wilcoxon signed-rank test and corrected using the Bonferroni method (α = 0.0167). Categorical data are presented as frequency counts (percentages), and the chi-square test and Fisher's exact test were used to assess differences. All analyses were performed using the SPSS statistical software (version 26.0. Armonk, NY, IBM Corp) and “anthro”, “anthroplus” and “MatchIt” packages in R (version 4.2.3). Statistical significance was set at *P* < 0.05.

## 3 Result

### 3.1 Baseline characteristics of the participants

A total of 2,210 adolescents aged 11–18 in the survey, including 1,125 (50.90%) males and 1,085 (49.10%) females, with 1,383 (62.58%) in the Junior High School group and 827 (37.42%) in the high school group. The mean age of the participants was 15.8 ± 1.6 years old. The overall overweight rate (1 < BMI *Z*-score ≤ 2) was 13.12% (290/2,210), and the overall obesity rate (BMI *Z*-score > 2) was 7.01% (155/2,210), with a combined proportion of 20.13%. The overall normal weight rate (−2 < BMI *Z*-score < 1) was 76.02% (1,680/2,210) and the overall emaciation rate (BMI *Z*-score ≤ −2) was 3.85% (85/2,210). The results are shown in Table 1.

**Table 1:** Basic characteristics of study participants.

Variables (*n* = 2,210)	Variable value
Age (year, Mean ± SD)	15.8 ± 1.6
Gender-Male (*n*, %)	1,125 (50.90)
Gender-Female (*n*, %)	1,085 (49.10)
Junior high school group (*n*, %)	1,383 (62.58)
High school group (*n*, %)	827 (37.42)
BMI *Z*-score [M(IQR)]	−0.13 (1.65)
BMI *Z*-score group (*n*, %)	
Obesity (BMI *Z*-score > 2)	155 (7.01)
Overweight (1 ≤ BMI *Z*-score ≤ 2)	290 (13.12)
Normal weight (−2 < BMI *Z*-score < 1)	1,680 (76.02)
Emaciation (BMI *Z*-score ≤ −2)	85 (3.85)

### 3.2 Comparison of BMI *Z*-score stratification by sex and grade levels

There was no significant difference in the overall BMI *Z*-score between males and females (*P* = 0.729). However, the distribution of BMI stratification differed significantly between sexes. The prevalence of obesity was higher in males than in females (χ^2^ = 13.556, *P* < 0.001), and the rate of emaciation was also higher in males (χ^2^ = 6.914, *P* = 0.009). The proportion of normal weight was significantly higher in females than in males (χ^2^ = 22.129, *P* < 0.001). No significant difference was observed in the prevalence of overweight between the sexes (χ^2^ = 2.432, *P* = 0.119; Table 2).

**Table 2:** Comparison of BMI *Z*-score between sex.

BMI stratification	Male (*n* = 1,125)	Female (*n* = 1,085)	*z*/χ^2^	OR (95%CI)	*P*
BMI *Z*-score	−0.14 (1.92)	−0.12 (1.34)	−0.347	–	0.729
Obesity (BMI *Z*-score > 2)	101 (8.98)	54 (4.98)	13.556	1.883 (1.338 ~ 2.650)	< 0.001^*^
Overweight (1 ≤ BMI *Z*-score ≤ 2)	160 (14.22)	130 (11.98)	2.432	1.218 (0.950 ~ 1.561)	0.119
Normal weight (−2 < BMI *Z*-score < 1)	808 (71.82)	872 (80.37)	22.129	0.623 (0.853 ~ 0.759)	< 0.001^*^
Emaciation (BMI *Z*-score ≤ −2)	56 (4.98)	29 (2.67)	7.935	1.908 (1.209 ~ 3.011)	0.005^*^

^*^Comparison between two groups, *P* < 0.05.

In the junior high school group, the overweight rate was 15.26% (211/1,383) and the obesity rate was 8.17% (113/1,383), with a combined proportion of 23.43%. In the senior high school group, the overweight rate was 9.55% (79/827), and the total obesity rate was 5.08% (42/827), with a combined proportion of 14.63%. The BMI *Z*-score differed significantly between the junior high school and high school groups, with the junior high school group showing higher values than the high school group (*P* < 0.001). The distribution of BMI *Z*-score stratification also varied significantly across grade levels. The prevalence of obesity was higher in the junior high school group than in the high school group (χ^2^ = 7.587, *P* = 0.006), and the overweight rate followed a similar pattern (χ^2^ = 10.945, *P* = 0.001). The proportion of normal weight was significantly higher in the high school group than in the junior high school group (χ^2^ = 11.55, *P* = 0.001). No significant difference was observed in the emaciation rate between the two groups (χ^2^ = 2.703, *P* = 0.100; Table 3).

**Table 3:** Comparison of BMI *Z*-score between grade levels.

BMI stratification	Junior high school group (*n* = 1,383)	High school group (*n* = 827)	*z*/χ^2^	OR (95%CI)	*P*
BMI *Z*-score [M(IQR)]	0.03 (1.69)	−0.34 (1.50)	−6.851	–	< 0.001^*^
Obesity (BMI *Z*-score > 2)	113 (8.17)	42 (5.08)	7.587	1.663 (1.154 ~ 2.396)	0.006^*^
Overweight (1 ≤ BMI *Z*-score ≤ 2)	211 (15.26)	79 (9.55)	10.945	1.705 (1.296 ~ 2.243)	0.001^*^
Normal weight (−2 < BMI *Z*-score < 1)	1,013 (74.55)	667 (80.65)	11.55	0.657 (0.533 ~ 0.810)	0.001^*^
Emaciation (BMI *Z*-score ≤ −2)	46 (3.33)	39 (4.72)	2.703	0.695 (0.450 ~ 1.075)	0.100

^*^Comparison between two groups, *P* < 0.05.

### 3.3 Results of lifestyle and dietary behavior survey

A total of 2,210 questionnaires were distributed in this survey, and 2,200 were recovered, with a questionnaire recovery rate of 99.55%. 10 questionnaires were lost in the non-obese group.

#### 3.3.1 Dietary behavior differences

Comparison between the obese and non-obese groups showed that there were more males than females in the obese group (χ^2^ = 13.899, *P* < 0.001), and the non-obese group was more likely to consider their diet structure reasonable (χ^2^ = 9.494, *P* = 0.002); however, the non-obese group also had a higher prevalence of picky eating habits (χ^2^ = 4.927, *P* = 0.026) and ate meals at home more frequently (χ^2^ = 38.524, *P* < 0.001). Moreover, the obese group consumed milk/dairy products as midnight snacks at a significantly higher rate than the non-obese group (χ^2^ = 7.453, *P* = 0.006).

#### 3.3.2 Differences in eating habits

The obese group exhibited significantly lower consumption rates of sweet foods (χ^2^ = 15.199, *P* < 0.001), vegetables (χ^2^ = 5.511, *P* = 0.019), fruits (χ^2^ = 10.742, *P* = 0.001), and “happy snacks” (χ^2^ = 8.353, *P* = 0.003) than the non-obese group. Regarding snack-related behaviors, the obese group consumed snacks between meals at a significantly higher rate (41.29 vs. 33.69%, χ^2^ = 4.346, *P* = 0.037), whereas they reported significantly lower rates of having no fixed snack time (44.52 vs. 54.03%, χ^2^ = 5.245, *P* = 0.022).

#### 3.3.3 Parental obesity

The obese group exhibited markedly higher proportions of paternal obesity (34.84 vs. 20.10%, χ^2^ = 29.714, *P* < 0.001), maternal obesity (30.32 vs. 17.02%, χ^2^ = 17.315, *P* < 0.001), and dual parental obesity (14.84 vs. 8.51%, χ^2^ = 7.082, *P* = 0.008) than the non-obese group. These findings indicate that parental obesity is an important factor influencing obesity among middle school students.

#### 3.3.4 Physical activity and sleep

Significant disparities were observed between the groups in terms of physical activity and sleep patterns. The obese group demonstrated lower daily exercise engagement, with a reduced proportion of participants exercising for approximately 1 h per day (65.16 vs.73.55%, χ^2^ = 10.186, *P* = 0.017). Notably, sleep-related behaviors also differed significantly: the obese group exhibited earlier bedtime patterns, characterized by a higher proportion of retiring before 10 p.m. (42.58 vs. 34.38%) but a lower proportion between 10–11 p.m. (36.77 vs. 46.45%, χ^2^ = 10.647, *P* = 0.031), alongside extended nocturnal sleep duration exceeding 8 h (34.84 vs. 24.79%) and reduced short sleep prevalence of 4–6 h (15.48 vs. 21.71%, χ^2^ = 8.724, *P* = 0.013).

#### 3.3.5 Health perception and psychological status

The obese group exhibited significantly greater exposure to passive smoking, with a higher proportion of participants reporting almost daily exposure (χ^2^ = 9.721, *P* = 0.021). Furthermore, marked disparities in body image perception were observed between groups (χ^2^ = 50.789, *P* < 0.001), wherein the obese group demonstrated diminished indifference toward body shape and substantially elevated rates of severe body dissatisfaction characterized by very low self-esteem and lack of confidence, suggesting profound psychological ramifications associated with obesity during adolescence. Further details are provided in Table 4.

**Table 4:** Comparison of lifestyle and dietary habits between obese and non-obese students.

Question	Obese group (*n* = 155)		Non-obese group (*n* = 2,045)		OR	95%CI
	*n*	(M%)	*n*	(M%)		
Gender (M/F)	101/54	65.16	1,015/1,030	49.63	1.898	1.349 ~ 2.671^*^
Are you concerned about your health? (Yes)	147	94.84	1,988	97.21	0.527	0.247 ~ 1.125
Do you think diet has an impact on your health? (Yes)	134	86.08	1,830	89.48	0.750	0.463 ~ 1.213
Is the diet structure reasonable? (Yes)	98	63.23	1,524	74.52	0.588	0.418 ~ 0.827^*^
Are you willing to change your bad eating habits? (Yes)	137	88.39	1,782	87.14	1.123	0.676 ~ 1.867
Do you have a picky eating habit? (Yes)	73	47.10	1,151	56.28	0.691	0.498 ~ 0.959^*^
Do you eat breakfast?						
Never eat	1	0.65	14	0.68	0.942	0.123 ~ 7.211
Sometimes eat	28	18.06	294	14.38	1.313	0.856 ~ 2.013
Eat frequently	37	23.87	450	22.00	1.111	0.757 ~ 1.631
Eat every day	89	57.42	1,287	62.93	0.794	0.571 ~ 1.106
Ways to eat lunch						
Go home and eat	19	12.26	282	13.79	1.000	–
Canteen	136	87.74	1,763	86.21	1.145	0.697 ~ 1.881
Ways to eat dinner						
Go home and eat	18	11.61	740	36.19	1.000	–
Canteen	137	88.39	1,305	63.81	4.316	2.619 ~ 7.113^*^
Like to eat food sold on the roadside outside the school						
Like	89	57.42	1,288	62.98	1.000	–
Dislike	66	42.58	757	37.02	1.262	0.906 ~ 1.756
Eat a midnight snack						
Never eat	51	32.90	524	25.62	1.423	1.004 ~ 2.019^*^
Sometimes eat	90	58.06	1,336	65.33	0.735	0.527 ~ 1.024
Eat frequently	10	6.45	148	7.24	0.884	0.456 ~ 1.714
Eat every day	4	2.58	37	1.81	1.438	0.506 ~ 4.087
Types of midnight snacks						
Fruits	83	53.55	1,066	52.13	1.059	0.763 ~ 1.469
Dried fruits	13	8.39	218	10.66	0.767	0.427 ~ 1.377
BBQ/Meat	58	37.42	780	38.14	0.970	0.692 ~ 1.359
Milk/dairy products	50	32.26	463	22.64	1.627	1.144 ~ 2.314^*^
Usual eating habits						
Vegetable	90	58.06	1,376	67.29	0.673	0.483 ~ 0.938^*^
Sweets	70	45.16	1,249	61.08	0.525	0.378 ~ 0.729^*^
Happy snacks	78	50.32	1,269	62.05	0.619	0.447 ~ 0.859^*^
Fruit	117	75.48	1,745	85.33	0.529	0.360 ~ 0.779^*^
Meat	109	70.32	1,476	72.18	0.913	0.639 ~ 1.307
When to eat snacks						
Do not eat	17	10.97	155	7.58	1.502	0.884 ~ 2.551
No time limit	69	44.52	1,105	54.03	0.683	0.491 ~ 0.948^*^
Between meals	65	41.29	689	33.69	1.421	1.020 ~ 1.981^*^
Before bed	12	7.10	102	4.99	1.599	0.858 ~ 2.977
Types of snacks						
Fried or puffed	69	44.52	1,019	49.83	0.808	0.582 ~ 1.122
Biscuits	61	39.35	733	35.84	1.162	0.831 ~ 1.623
Preserved fruits	49	31.61	576	28.17	1.179	0.829 ~ 1.676
Total exercise time per week						
< 2.5 h	90	58.06	1,054	51.54	1.000	–
>2.5 h	65	41.94	991	48.46	0.768	0.552 ~ 1.069
Average daily sitting time						
< 90 min	73	47.10	910	44.50	1.000	–
>90 min	82	52.90	1,135	55.50	0.901	0.649 ~ 1.249
Interest in outdoor sports activities						
Not interested	31	20.00	307	15.01	1.000	–
Interested in actively participating	124	80.00	1,738	84.99	0.707	0.468 ~ 1.067
Physical exercise time						
Less than 1 h per week	5	3.23	41	2.00	1.629	0.634 ~ 4.184
About 1 h per week	14	9.03	86	4.21	2.262	1.254 ~ 4.081^*^
3 days and 1 h	35	22.58	414	20.24	1.149	0.777 ~ 1.700
About 1 h per day	101	65.16	1,504	73.55	0.673	0.477 ~ 0.950^*^
How many times a week do you engage in sports?						
Never	4	2.58	33	1.61	1.615	0.565 ~ 4.619
Once a week	10	6.45	128	6.26	1.033	0.531 ~ 2.009
2–3 times a week	85	54.84	1,005	49.14	1.257	0.905 ~ 1.744
More than 4 times a week	56	36.13	879	42.98	0.750	0.534 ~ 1.053
^*^Nighttime bedtime						
Before 10 o'clock	68	42.58	703	34.38	1.492	1.073 ~ 2.076^*^
10:00–11:00	55	36.77	950	46.45	0.634	0.451 ~ 0.891^*^
After 11 o'clock	20	12.90	294	14.38	0.882	0.543 ~ 1.434
After 12 o'clock	10	6.45	75	3.67	1.811	0.917 ~ 3.579
After 1 a.m.	2	1.29	23	1.12	1.149	0.268 ~ 4.920
Daily sleep time						
More than 8 h	54	34.84	507	24.79	1.622	1.148 ~ 2.291^*^
6–8 h	77	49.68	1,094	53.50	0.858	0.619 ~ 1.190
4–6 h	24	15.48	444	21.71	0.661	0.422 ~ 1.034
Time spent using electronic products every day						
None	16	10.32	319	15.60	0.623	0.366 ~ 1.059
Less than 1 h	45	29.03	557	27.24	1.093	0.762 ~ 1.567
2–3 h	64	41.29	805	39.36	1.083	0.777 ~ 1.510
More than 4 h	30	19.35	364	17.80	1.108	0.732 ~ 1.677
Daily transportation to and from school						
Walk	40	25.81	505	24.69	1.061	0.730 ~ 1.541
Bike	6	3.87	50	2.44	1.607	0.678 ~ 3.808
School bus/private car/public bus	109	70.32	1,490	72.86	0.883	0.617 ~ 1.263
Average number of days per week of passive smoking						
0 days	141	90.97	1,917	93.74	0.672	0.377 ~ 1.198
1–2 days	6	3.87	84	4.11	0.940	0.404 ~ 2.188
3–5 days	1	0.65	17	0.83	0.775	0.102 ~ 5.860
Almost every day	7	4.52	27	1.32	3.535	1.514 ~ 8.253^*^
Parental obesity						
Both father and mother are obese	23	14.84	174	8.51	1.874	1.172 ~ 2.996^*^
Father is obese	54	34.84	411	20.10	2.126	1.501 ~ 3.009^*^
Maternal obesity	47	30.32	348	17.02	2.122	1.479 ~ 3.046^*^
Personality and mental state						
Taciturn	8	5.16	128	6.26	0.815	0.391 ~ 1.698
Moderate	77	49.68	1,054	51.54	0.928	0.670 ~ 1.287
Enthusiastic and cheerful	70	45.16	863	42.20	1.128	0.812 ~ 1.566
Attitude towards one's own body shape (fat or thin, etc.)						
It doesn't matter	42	27.10	1,036	50.66	0.362	0.251 ~ 0.521^*^
A bit inferior	78	50.32	839	41.03	1.456	1.050 ~ 2.019^*^
Very low self-esteem and lack of confidence	35	22.58	170	8.31	3.217	2.139 ~ 4.837^*^
Whether nocturnal emission has occurred (for men) or menarche (for women)						
No	67	43.23	895	43.77	1.000	–
Yes	88	56.77	1,150	56.23	1.022	0.735 ~ 1.421
Whether there are morning erections (for men) or irregular menstruation (for women)						
No	100	64.52	1,459	71.34	1.000	–
Yes	55	35.48	586	28.66	1.369	0.972 ~ 1.929

^*^P < 0.05 compared with non-obese group.

### 3.4 Effects of the health-education intervention

This study conducted a 6-month intervention follow-up among 155 obese middle school students, with 114 (73.55%) students completing the 3-month follow-up and 111 (71.61%) students completing the 6-month follow-up.

#### 3.4.1 Improvement in health awareness

Students' awareness of the relationship between diet and health improved significantly following the intervention. The proportion of students who believed that diet affects health increased from 86.45% at baseline to 97.37% at 3 months and 94.59% at 6 months, with a statistically significant difference (χ^2^ = 12.102, *P* = 0.002), indicating that the intervention effectively enhanced students' healthy eating awareness.

#### 3.4.2 Changes in dietary behavior

Significant improvements were observed in the choice of midnight snacks. The proportion of students selecting meat and dairy products as midnight snacks decreased from 69.68% at baseline to 51.75% at 3 months and 46.85% at 6 months, with a statistically significant difference (χ^2^ = 16.111, *P* < 0.001), suggesting that the intervention effectively reduced the consumption of high-fat and high-protein midnight snacks.

#### 3.4.3 Enhancement of exercise habits

The duration of physical exercise significantly increased. The proportion of students exercising approximately 1 h per day rose from 65.16% at baseline to 79.82% at 3 months and was maintained at 74.77% at 6 months, with a statistically significant difference (χ^2^ = 7.517, *P* = 0.023), demonstrating that the intervention effectively promoted daily physical activity among the students.

#### 3.4.4 Alterations in sleep patterns

The daily sleep duration was significantly decreased. The proportion of students sleeping for more than 8 h declined from 34.84% at baseline to 25.44% at 3 months and 21.62% at 6 months, with a statistically significant difference (χ^2^ = 6.181, *P* = 0.045), indicating that the intervention may have helped adjust students' sleep duration to a more appropriate range.

No significant changes were observed in other behaviors or habits before or after the intervention period. The details are presented in Table 5.

**Table 5:** Changes in lifestyle and dietary behaviors of obese middle school students before and after intervention (*n*, %).

Question	Before intervention (*n* = 155)		3-month intervention (*n* = 114)		6-month intervention (*n* = 111)		χ^2^	*P*
	*n*	%	*n*	%	*n*	%		
Are you concerned about your health? (Yes)	147	94.84	109	95.61	103	92.79	0.929	0.630
Do you think diet has an impact on your health? (Yes)	134	86.45	111	97.37	105	94.59	12.102	0.002^*^
Is the diet structure reasonable? (Yes)	98	63.23	78	68.42	82	73.87	3.385	0.184
Are you willing to change your bad eating habits? (Yes)	137	88.39	98	85.96	99	89.19	0.609	0.737
Do you have a picky eating habit? (Yes)	73	47.10	58	50.88	57	51.35	0.597	0.742
Do you eat breakfast (eat every day + eat often)	126	81.29	87	76.32	83	74.77	1.830	0.400
How to eat lunch (eat at home)	19	12.26	20	17.54	18	16.22	1.621	0.445
How to eat dinner (eat at home)	19	12.26	22	19.30	15	13.51	2.778	0.249
Like to eat food sold by street vendors outside school (Like)	89	57.42	64	56.14	57	51.35	1.014	0.602
Eating midnight snacks (every day + often)	14	9.03	10	8.77	9	8.11	0.071	0.965
Types of midnight snacks (meat + dairy)	108	69.68	59	51.75	52	46.85	16.111	< 0.001^*^
Usual eating habits (meat)	109	70.32	79	69.30	82	73.87	0.640	0.726
When to eat snacks (before bedtime + any time)	81	52.26	54	47.37	56	50.45	0.630	0.730
Interest in outdoor sports activities (no interest)	31	20.00	16	14.04	17	15.32	1.930	0.381
Physical exercise time (about 1 h per day)	101	65.16	91	79.82	83	74.77	7.517	0.023^*^
How many times a week do you engage in physical activity (≥4 times a week)	56	36.13	42	36.84	37	33.33	0.344	0.842
Bedtime at night (before 10 o'clock)	56	36.13	38	33.33	40	36.04	0.266	0.875
Daily sleep time (more than 8 h)	54	34.84	29	25.44	24	21.62	6.181	0.045^*^
Daily time spent using electronic products (less than 1 h)	45	29.03	39	34.21	44	39.64	3.278	0.194
Daily transportation to and from school (bicycle + walking)	46	29.68	37	32.46	28	25.23	1.454	0.484

^*^P < 0.05, the difference was statistically significant.

### 3.5 Changes in BMI *Z*-score and biochemical indicators

At the 6-month follow-up, complete blood lipid data were obtained from 111 adolescents with obesity. Forty-four participants were lost to follow-up (reasons at 3 months: sick/personal leave, *n* = 11; competition participation, *n* = 10; school transfer, *n* = 2; reasons at 6 months: sick/personal leave, *n* = 6; refusal of blood collection, *n* = 8; hemolyzed sample, *n* = 1; unreturned questionnaires, *n* = 6). There were no statistically significant differences in gender, grade, age, or baseline biochemical indicators between the 44 lost-to-follow-up participants and the 111 individuals who completed the 6-month follow-up (*P* > 0.05), indicating that the 6-month sample remained demographically representative of the original cohort. The details are presented in Table 6.

**Table 6:** Comparison of baseline characteristics between completers and the lost-to-follow-up participants.

Variables	Completers *n* = 111	Lost to follow-up *n* = 44	χ^2^/*Z/t*	*P*
Age	16.02 ± 3.22	14.93 ± 3.41	1.869	0.064
Gender (M/F)	66/45	35/9	2.923	0.087
BMI *Z*-score	2.22 ± 0.06	2.20 ± 0.07	1.783	0.077
Fasting blood sugar (mmol/L)	4.98 ± 0.03	4.94 ± 0.05	1.528	0.129
Triglycerides (mmol/L)	0.97 (0.61)	1.07 (0.63)	−1.484	0.138
Total cholesterol (mmol/L)	3.67 ± 0.63	3.65 ± 0.61	0.180	0.858
HDL cholesterol (mmol/L)	1.02 ± 0.16	1.00 ± 0.15	0.714	0.476
LDL cholesterol (mmol/L)	2.24 ± 0.59	2.20 ± 0.61	0.377	0.707
Insulin (μU/mL)	11.78 (6.73)	11.46 (8.48)	−0.401	0.689
TyG	8.27 ± 0.46	8.21 ± 0.51	0.710	0.479
HOMA-IR	2.57 (1.53)	2.74 (1.18)	−1.016	0.310

After the intervention, this study collected BMI *Z*-score data from 111 obese individuals 6 months after the two health education interventions to evaluate their effectiveness of health education. The results showed that the overall population exhibited a trend of initial decrease followed by a rebound in BMI *Z*-score; however, the time effect was not statistically significant (*P* = 0.935). However, in males, the change in BMI *Z*-score over time (baseline, 3 months, and 6 months) was statistically significant (*P* = 0.001). In contrast, the changes in BMI *Z*-score in females and the different grade subgroups were not statistically significant (*P* > 0.05), indicating that the two health education interventions failed to produce sustained and effective weight control (Table 7).

**Table 7:** Changes in BMI *Z*-score of obese individuals after two health education interventions (*n* = 111).

Grouping	First BMI *Z*-score	3 months BMI *Z*-score	6 months BMI *Z*-score	*F*/χ^2^	*P*
Overall	2.22 ± 0.06	2.20 ± 0.07	2.23 ± 0.08	0.067	0.935
Gender					
Male (*n* = 66)	2.34 ± 0.61	2.17 ± 0.64^*^	2.28 ± 0.69^Δ^	7.458	0.001
Female (*n* = 45)	2.28 (0.60)	2.22 (0.77)	2.35 (0.55)	5.485	0.064
Grade					
Junior high school (*n* = 71)	2.36 (0.50)	2.30 (0.62)	2.39 (0.58)	0.932	0.628
High school (*n* = 40)	1.95 (0.83)	1.92 (0.70)	2.05 (0.55)	0.348	0.840

^*^P < 0.05 compared with before intervention; Δ*P* < 0.05 compared with 3 months after intervention.

After 6 months of intervention, among 111 adolescents with obesity, significant main effects of time for fasting blood glucose, total cholesterol, and LDL cholesterol were identified (*P* < 0.01). *Post-hoc* tests revealed that fasting blood glucose, total cholesterol, and LDL cholesterol levels were significantly reduced at 3 and 6 months post-intervention compared to baseline (all *P* < 0.05). Furthermore, LDL cholesterol levels showed an additional significant decrease at 6 months compared to those at 3-month time point (*P* < 0.05; Table 8).

**Table 8:** Changes in blood lipid-related indicators before and after health intervention in obese children (*n* = 111).

Blood lipid-related indicators	Before intervention	3 months after intervention	6 months after intervention	*F*/χ^2^	*P*
Fasting blood sugar (mmol/L)	4.98 ± 0.03	4.60 ± 0.04^*^	4.62 ± 0.03^*^	44.267	< 0.001
Triglycerides (mmol/L)	0.97 (0.61)	0.99 (0.49)	1.01 (0.64)	1.430	0.489
Total cholesterol (mmol/L)	3.67 ± 0.63	3.52 ± 0.64^*^	3.42 ± 0.62^*^	5.835	0.009
HDL cholesterol (mmol/L)	1.02 ± 0.16	0.98 ± 0.15	0.97 ± 0.16	1.519	0.223
LDL cholesterol (mmol/L)	2.24 ± 0.59	2.04 ± 0.46^*^	1.97 ± 0.44^*Δ^	11.129	< 0.001
Insulin (μU/mL)	11.78 (6.73)	10.79 (6.46)	11.12 (6.47)	1.503	0.472
TyG	8.27 ± 0.46	8.22 ± 0.43	8.20 ± 0.44	0.878	0.391
HOMA-IR	2.57 (1.53)	2.19 (1.43)	2.29 (1.37)	4.667	0.097
Dyslipidemia^a^ (*n*, %)	94 (84.68)	81 (72.97)	81 (72.97)	5.710	0.058
Triglycerides	20 (18.02)	15 (13.51)	17 (15.32)	0.866	0.649
Total cholesterol	5 (4.50)	1 (0.90)	0 (0.00)	–	0.019^▴^
HDL cholesterol	64 (66.67)	65 (61.26)	64 (66.67)	0.025	0.988
LDL cholesterol	5 (4.50)	0 (0.00)^*^	0 (0.00)^*^	–	0.012^▴^

^*^P < 0.05 compared with before intervention; Δ*P* < 0.05 compared with 3 months after intervention; ▴Fisher's exact test.

a: dyslipidemia was defined as patients with total cholesterol ≥ 5.2 mmol/L or triglycerides ≥1.5 mmol/L or HDL-c < 1.0 mmol/L or LDL-c ≥3.4 mmol/L.

Analysis of categorical variables showed that the prevalence of abnormal LDL cholesterol declined from 4.50% at baseline to 0% after the intervention (*P* = 0.012), while abnormal total cholesterol decreased from 4.50 to 0% (*P* = 0.019). The overall prevalence of dyslipidemia exhibited a marginally significant downward trend from 84.68 to 72.97% (*P* = 0.058). In contrast, no significant changes were observed in triglyceride levels, fasting insulin levels, TyG index, or HOMA-IR during the intervention period (all *P* > 0.05). See Table 8.

## 4 Discussion

The results of this study show that the rates of overweight and obesity among adolescents in the farming and pastoral areas of Inner Mongolia, China, are relatively high, with the rate of overweight exceeding the national average (2). The combined prevalence of overweight and obesity was 20.13%. Among them, the overweight and obesity rates in the junior high school group were higher than those in the senior high school group. The questionnaire survey revealed that compared to the obese group, the non-obese group: (1) had a higher perception that their diet was balanced; (2) had a higher proportion of eating at home than at school; (3) spent more time on physical exercise; (4) majority controlled their sleep time to 6–8 h; and (5) had a lower proportion of parental obesity, suggesting that adolescents with obesity have a significant hereditary predisposition. Consistent with the results of the COVID-19 pandemic, changes in eating behaviors, increased food intake, and restricted physical activity due to lockdown measures have led to a significant increase in childhood and adolescent obesity rates in many countries (18, 19). In recent years, the significant increase in overweight and obesity rates among adolescents has also been related to smartphone use, late bedtimes, and excessive consumption of sugary drinks (20–23). The higher overweight and obesity rates among Mongolian adolescents are more closely related to their dietary habits and family histories.

Following Mongolian–Chinese bilingual health education, we conducted two consecutive dynamic observations (once every 3 months) of biochemical indicators and dietary behavior changes among obese Mongolian adolescents in agro-pastoral areas. In our study of 111 obese adolescents, we found that male BMI *z*-scores significantly decreased at 3 months of intervention but rebounded at 6 months, showing no significant difference from baseline. This suggests that the current health education intervention yielded immediate positive outcomes for obese male adolescents. This initial success may be attributed to robust motivation and higher compliance during the early phases, indicating that short-term interventions are both feasible and effective in this demographic. However, the rebound in BMI *Z*-scores at the 6-month follow-up highlights the challenge of sustaining these benefits. This trajectory of “short-term decline followed by long-term rebound” is frequently observed in adolescent weight management and likely reflects two underlying factors: the inherent instability of adolescent behavioral habits, which complicates the long-term adherence to healthy dietary and exercise regimens post-intervention, and the physiological weight gain associated with male pubertal development, suggesting that standalone health education is insufficient to counteract these natural growth trajectories over time. In addition, preliminary improvements in metabolic indicators were observed. Blood glucose, TC, and LDL-C levels improved significantly compared to the baseline, and the prevalence of dyslipidemia (specifically elevated TC and LDL-C) was reduced. This indicates that the intervention effectively lowered these two core risk factors for atherosclerosis in adolescents with obesity, thereby directly decreasing the cardiometabolic risk. In terms of dietary behaviors, the time spent on physical exercise increased significantly, and excessive sleep time was reduced. This is consistent with previous research: among adolescents, lifestyle interventions can reduce systemic inflammation and metabolic disorders associated with overweight/obesity and significantly lower lipid and glucose levels, even in the absence of marked BMI reduction.

Health literacy is a prerequisite for effective health intervention. Among Mongolian adolescents, language barriers inherent in monolingual Chinese interventions impede accurate comprehension of obesity prevention knowledge, leading to low health cognition and poor compliance. To address this, the present study designed a Mongolian–Chinese bilingual intervention that precisely dismantles these linguistic and cultural barriers. This ensures the accurate acquisition and deep processing of health information, thereby increasing adolescents' health literacy. Evidence from culturally and linguistically diverse (CALD) populations corroborates this, demonstrating that language-matched interventions significantly boost health literacy in vulnerable groups and act as a core pathway for improving long-term health outcomes (24).

Guided by the Health Belief Model (HBM) and the Theory of Planned Behavior (TPB), behavioral change hinges on perceived benefits and intentions. Traditional interventions often cause linguistic and cultural alienation, leaving adolescents with low perceived behavioral control and weak health intentions. The proposed bilingual intervention mitigates this by incorporating culturally congruent guidance that fosters trust and cultural appropriateness. Consequently, it lowers perceived barriers, heightens the perceived benefits of dietary modification, and shapes positive behavioral attitudes and subjective norms, validating the utility of behavioral change theories in explaining the intrinsic mechanisms of bilingual interventions (24, 25).

Exercise is essential for mitigating adolescent obesity and its comorbidities. Aerobic, resistance, and combined training equally reduce insulin resistance (26), and yield greater reductions in central obesity when paired with dietary changes (27). Because childhood obesity predisposes individuals to cardiovascular disease (CVD), engaging in moderate-to-vigorous physical activity (MVPA) is crucial for improving cardiorespiratory fitness and overall cardiovascular health across youths aged 6–17 (28, 29). Both acute school-based interventions like high-intensity interval training (HIIT) (30) and sustained programs (e.g., ≥9 months) (31) effectively lower overweight risks. Furthermore, prolonged structured exercise (e.g., combined aerobic-resistance training) significantly optimizes cardiometabolic profiles, including reducing BMI and LDL cholesterol (32).

In this study, the use of bilingual (Mongolian and Chinese) health education fully considered the actual language and cultural background of the Mongolian population in rural and pastoral areas, making the content of health education easier to accept and understand, and ensuring the effectiveness of information delivery. This highlights the importance of tailoring health education to the actual conditions. Health education must be closely integrated into real-world scenarios. Unique characteristics related to the region, culture, economy, and language exist in special environments, such as rural and pastoral areas. Therefore, when conducting health education, it is necessary to gain an in-depth understanding of the local situation, including living habits, dietary features, cultural traditions, and educational levels, and develop an education program that matches these conditions. For example, in terms of language, providing health education in a language familiar to local residents can remove language barriers, enhance communication, and improve their acceptance of health information. Regarding educational resources, appropriate educational methods and tools should be selected based on local economic circumstances and educational facilities, such as community outreach, home visits, and simple promotional materials, to ensure practical and effective implementation of health education. Multi-faceted interventions to promote healthy eating, physical activity, and supportive environments (encouraging the participation of schools and families to support adolescents' behavior change). These interventions also improved other obesity-related outcomes, dietary habits, sedentary behavior, physical activity, and knowledge about obesity. In different socioeconomic regions, multifaceted interventions have proven effective in preventing obesity among primary school adolescents (33). Adolescent obesity is related to adult obesity and a higher risk of chronic disease. School interventions focusing on nutritional education and physical activity are crucial for preventing obesity in childhood. School-based interventions are an effective long-term strategy for controlling obesity in childhood. Combined interventions involving physical activity (PA), health education (HE), and school policies (SP) are more effective in reducing BMI than interventions that address only one aspect (34). Studies have reported that multidisciplinary interventions (both family and individual) for overweight and obese adolescents, lasting 12 weeks (3 days per week, each session lasting 1 h and 30 min), with 30 min of theoretical intervention (nutrition education and psychological education) and 1 h of physical exercise, lead to significant reductions in body fat mass (FM), body fat percentage (BF), visceral fat, fasting blood glucose, TG, TC, LDL-c, and DBP after the intervention, while HDL-c levels increase (35, 36).

In recent years, most adolescent obesity research has relied on school-based interventions, with most programs focusing primarily on people-oriented educational approaches such as nutrition and dietary education courses. These are combined with promoting physical activity and healthy lifestyles among students, parents, and school staff, reducing exposure to unhealthy environments, and providing healthier food choices (37). Adolescent obesity is a multifactorial pathological process, and its management should involve patients, families, schools, and communities, and even require policy support from the government. Lifestyle modifications, including adopting a healthy diet and increasing physical activity, are the cornerstones of treatment (38).

### 4.1 Limitations

This study had certain limitations. First, there was no control group. Due to the lack of a concurrent control group, the improvements in biochemical indicators and changes in diet and exercise behaviors observed in this study cannot be fully attributed to the health education intervention. Adolescents are in a period of rapid growth and development, and their metabolic indicators may change naturally with age. In addition, seasonal factors (such as the warming climate during the intervention period, leading to an increase in outdoor activities) may have confounding effects on the results. Therefore, a randomized controlled trial design should be adopted in the future to confirm the effects of this intervention. Second, the intervention period was only 6 months, and the intervention frequency was low, which may not be sufficient to cause a significant change in the BMI *Z*-score. The lack of long-term follow-up data makes it impossible to evaluate the persistence of the intervention effects. Third, the sample size was relatively small, not covering normal weight and overweight populations, and cannot comprehensively evaluate the intervention effect of different weight classifications; it only comes from three schools in the same region, limiting the extrapolation of the results. Future research should conduct a prospective study with a large sample size, multiple centers, and long-term follow-up to further verify the effect of Mongolian–Chinese bilingual health education on the prevention and control of obesity among adolescents in ethnic minority areas. Fourth, self-reported behavioral data, diet, and physical activity assessments rely on self-report questionnaires, introducing potential recall and social expectation biases.

## 5 Conclusion

Bilingual health education led to initial improvements in dietary behaviors and metabolic indicators, particularly blood glucose and lipid levels, in adolescents with obesity. It also increased exercise duration and awareness of excessive sleep, leading to a short-term reduction in BMI *Z*-scores in male adolescents. These findings underscore the importance of tailoring health education to the linguistic and cultural backgrounds of the target populations. However, given the limited health education resources in rural pastoral areas, effective obesity prevention and control necessitates coordinated efforts from the government, schools and families. Despite these promising practical implications, this study has several limitations. The current findings are preliminary, exploratory results from a single-group intervention study. The absence of a control group, relatively short intervention period, and limited sample size restrict our ability to draw definitive causal inferences and generalize these findings. Therefore, future large-scale, multi-center, prospective randomized controlled trials with long-term follow-ups are warranted to conclusively verify the efficacy of this bilingual intervention in ethnic minority areas.

## Data Availability

The data cannot be fully disclosed due to ethical and privacy concerns. Readers may contact the corresponding author to obtain the dataset upon request.
